# Anodal Transcranial Direct Current Stimulation Shows Minimal, Measure-Specific Effects on Dynamic Postural Control in Young and Older Adults: A Double Blind, Sham-Controlled Study

**DOI:** 10.1371/journal.pone.0170331

**Published:** 2017-01-18

**Authors:** Chesney E. Craig, Michail Doumas

**Affiliations:** 1 School of Psychology, Queen’s University Belfast, Belfast, Co. Antrim, United Kingdom; 2 Research Centre for Health, Exercise and Active Living, Department of Exercise and Sport Science, Manchester Metropolitan University, Crewe, Cheshire, United Kingdom; University Medical Center Goettingen, GERMANY

## Abstract

We investigated whether stimulating the cerebellum and primary motor cortex (M1) using transcranial direct current stimulation (tDCS) could affect postural control in young and older adults. tDCS was employed using a double-blind, sham-controlled design, in which young (aged 18–35) and older adults (aged 65+) were assessed over three sessions, one for each stimulatory condition–M1, cerebellar and sham. The effect of tDCS on postural control was assessed using a sway-referencing paradigm, which induced platform rotations in proportion to the participant’s body sway, thus assessing sensory reweighting processes. Task difficulty was manipulated so that young adults experienced a support surface that was twice as compliant as that of older adults, in order to minimise baseline age differences in postural sway. Effects of tDCS on postural control were assessed during, immediately after and 30 minutes after tDCS. Additionally, the effect of tDCS on corticospinal excitability was measured by evaluating motor evoked potentials using transcranial magnetic stimulation immediately after and 30 minutes after tDCS. Minimal effects of tDCS on postural control were found in the eyes open condition only, and this was dependent on the measure assessed and age group. For young adults, stimulation had only offline effects, as cerebellar stimulation showed higher mean power frequency (MPF) of sway 30 minutes after stimulation. For older adults, both stimulation conditions delayed the increase in sway amplitude witnessed between blocks one and two until stimulation was no longer active. In conclusion, despite tDCS’ growing popularity, we would caution researchers to consider carefully the type of measures assessed and the groups targeted in tDCS studies of postural control.

## Introduction

Postural control is an adaptive sensorimotor process involving constant integration of sensory information from three channels; visual, somatosensory (proprioceptive) and vestibular. Information from these channels is integrated using a sensory reweighting process [1], under which the weight of each channel is determined by the channel’s relative reliability, in order to obtain the most accurate percept of the current postural state. Previous experimental evidence suggests that sensory reweighting is slower in older adults [2–4]. For example, older adults are more likely to fall if they experience a conflict in any channel (visual or proprioceptive) of sensory information compared to young adults [2], especially within the first trial. Although sensory reweighting mechanisms have been highlighted as a contributor to the high prevalence of falls in older adults [5,6], the literature on this issue is limited, especially in terms of the neural mechanisms underlying this process. The present study aimed to investigate the role of the cerebellum and the primary motor cortex in sensory reweighting in young and older adults’ postural control and whether brain stimulation over these areas could affect this process.

The cerebellum (particularly its anterior lobe) is considered critical for postural coordination [7] because it is one of the main brain regions associated with sensorimotor integration [8] and it receives substantial input from the three sensory channels involved in postural control. A recent study by Pijnenburg et al. [9] supported the role of cerebellum in sensory reweighting, specifically in *proprioceptive* reweighting. The study found that degraded white matter integrity in the superior cerebellar peduncle was associated with diminished proprioceptive reweighting abilities in patients with lower back pain. Similarly, a study using galvanic vestibular stimulation [10] showed that over a 7–8 week period, the postural response to bilateral bipolar galvanic vestibular stimulation successfully returned to baseline, however, the vestibular-ocular and spinal reflexes did not change. This suggests that the vestibular input did not adapt but rather the weighting of this channel must have been modified, which the authors postulate may occur within the cerebellum. Similarly, Guo and Raymond [11] implicated the cerebellum in sensory reweighting using a cerebellum-dependent learning paradigm in which monkeys reduced the variability of their smooth eye movements to compound visual-vestibular stimuli. This was attributed to up-weighting of the vestibular channel which was less variable than the visual channel in this paradigm.

Although the cerebellum has been associated with sensory reweighting using brain imaging [9] and cerebellum-dependent learning paradigms [11], a technique that could elucidate whether cerebellar activity plays a *causal* role in sensory reweighting and is not merely a by-product of activity in another region, is brain stimulation. Brain stimulation has been used extensively as a tool to establish the functional relevance of brain regions to motor behaviour and to facilitate activity in specific regions in order to improve motor outcomes [12–14]. tDCS is a low-cost neurostimulation technique that has received growing attention over the past decade, after it was demonstrated that tDCS over M1 affects motor evoked potentials (MEPs; [15]). tDCS uses a weak direct current (DC; ~1-3mA) applied to the scalp, typically for 15–20 minutes using two or more surface electrodes, to modulate cortical excitability in the underlying regions in a polarity-specific manner [16]. Even though the mechanisms of action of this technique are yet to be fully elucidated [17,18], its advantage is the ability to stimulate during task performance (online) and alter behaviour, thus clarifying whether the brain regions under stimulation are involved in a particular task.

The literature on the effects of tDCS on motor behaviour initially focused on the role of the primary motor cortex (M1), with anodal tDCS over M1 demonstrating enhanced corticospinal excitability up to 90 minutes after stimulation [15]. In terms of behavioural effects, this has been associated with enduring motor adaptation [19], which suggests that anodal M1 tDCS is associated with neurophysiological long-term potentiation and alters brain plasticity [20]. Following this, Jayaram et al. [21] investigated the role of the cerebellum in motor adaptation using anodal cerebellar tDCS on a locomotor adaptation task, namely split-belt treadmill walking. During this task, anodal cerebellar tDCS resulted in a faster rate of adaptation. This in contrast to the study by Kaski et al. (32), which found that anodal M1 tDCS resulted in longer-lasting locomotor adaptation. This evidence suggests that the M1 and cerebellum may have dissociated roles in motor adaptation. Galea and colleagues [22] examined this using anodal cerebellar and M1 tDCS during a visuomotor adaptation task and showed that cerebellar tDCS affects the rate of *acquisition* of locomotor adaptation tasks, whereas M1 tDCS affects the *retention* of locomotor adaptation aftereffects. Dutta, Paulus and Nitsche [23] reported contradictory findings to Galea et al. [22] in their study on the effects of online anodal tDCS on tracking performance in an EMG biofeedback visual pursuit task. They found that cerebellar tDCS instead slowed the rate of motor learning. However, the between-subjects design of this study led to a small sample size of only four participants per group. In light of these findings into the dissociated roles of the cerebellum and M1 in sensorimotor adaptation, the current study aimed to examine effects of cerebellar and M1 tDCS on postural control.

M1 is relevant to postural control, as it is included in the frontal cortico-basal ganglia network, which is thought to be involved in the control of gait and balance [24]. A study by Dutta & Chugh [25] showed that online anodal M1 tDCS for a duration of 10 minutes could improve postural stability during eyes closed quiet stance in young adults. This was accompanied by an increase in MEP-assessed corticospinal excitability. However, it is yet to be demonstrated whether tDCS could be used similarly to improve postural control in older adults. Most tDCS studies typically assess young participants [12,19], however, tDCS interventions may be particularly relevant to the ageing population, especially interventions involving tasks critical for daily life and independence, like postural control. Neural plasticity changes with age, contributing to reduced motor learning [26] and tDCS could provide a novel tool to preserve motor function in this age group [13,20,27,28]. For example, a recent study has shown that online anodal M1 tDCS (1mA for 20 minutes) can significantly improve the acquisition of a novel finger tapping sequence in older adults, with enhanced retention up to 24 hours later [27]. This was not witnessed in younger adults, who showed better baseline acquisition, however, this may be a ceiling effect. It has been suggested that future studies should modify task difficulty for different ages, in order to remove age effects at baseline [29]. This restoration of motor learning in older adults in response to online tDCS can also be witnessed in a study that demonstrated that visuomotor tracking error could be decreased by 12–22%, immediately after and 30 minutes after unilateral or bilateral M1 tDCS (1mA for 15 minutes; [20]). This effect was associated with increased motor evoked potentials (MEPs) and reduced short-interval intracortical inhibition [20]. More importantly, there is mounting evidence that tDCS can improve lower limb function and gait. This has been shown in locomotor adaptation in healthy young participants [19,21,22] and in motor/balance outcomes for patient groups, such as, after stroke [30], hemicerebellectomy [31], Parkinson’s disease [32,33], leukoaraiosis [34] and in children with cerebral palsy [35]. However, little is known about whether tDCS stimulation can alter sensory reweighting in healthy older adults’ postural control.

The aim of the present study was to examine the effect of anodal transcranial direct current stimulation (tDCS) over the cerebellum and primary motor cortex (M1) on postural responses during a sensory reweighting paradigm. This was achieved using a double-blinded, sham-controlled design in which young and older adults’ postural responses were evaluated over three separate sessions, one for each tDCS condition–M1, cerebellar and sham–which were counterbalanced. Postural control was assessed using a sway-referencing paradigm, in which the support surface tilted in proportion to the participant’s body sway, requiring the use of appropriate sensory reweighting [36,37]. Additionally, in line with Vallence and Goldsworhty’s [29] suggestion that task difficulty should be manipulated to prevent baseline age differences, the level of compliance of the support surface was higher for young adults. Postural control was assessed before, during, immediately after and 30 minutes after tDCS. Effects on corticospinal excitability were also measured prior to, immediately after and 30 minutes after tDCS. We hypothesised that anodal tDCS over either the M1 or the cerebellum would alter postural performance compared to sham tDCS.

## Methods

### Participants

**Fig 1:**
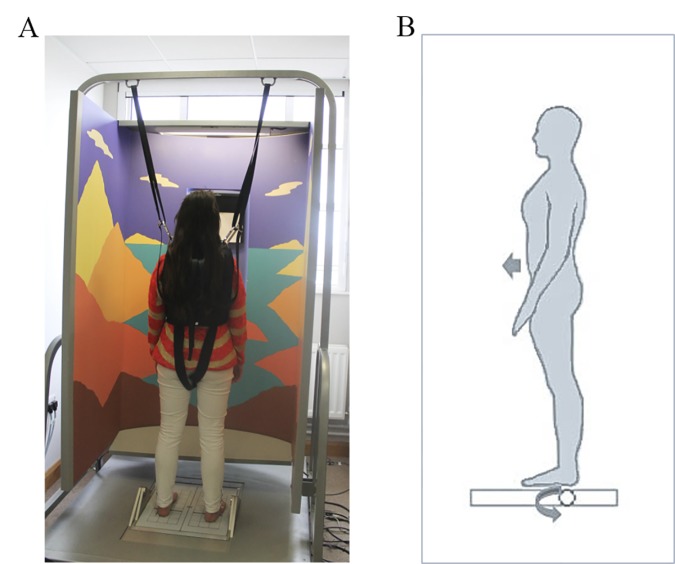
Image of Balance Master System, alongside a pictorial representation of the sway referencing technique. (A) Image of Balance Master Clinical Research System (NeuroCom International, Inc., Clackamas, OR, USA). (B)During sway referencing, the support surface rotates about the ankle axis in proportion to AP sway, making the main proprioceptive signal used in postural control inaccurate.

**Fig 2:**
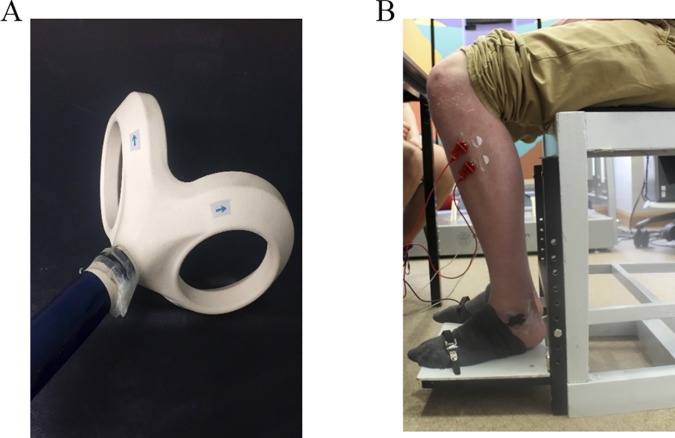
Image of the angled double cone coil and foot support. (A) Angled double cone coil (Magstim, Whitland, United Kingdom). (B) Foot support used for active dorsiflexion during TMS.

**Fig 3:**
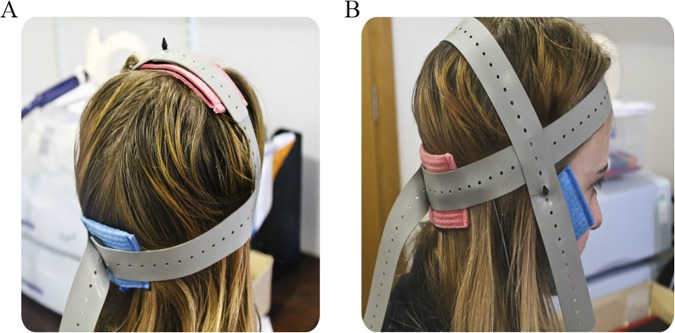
Electrode positions for M1 and cerebellar tDCS. (A) During M1 stimulation, the anode was placed on the leg area motor hotspot, as identified during single-pulse TMS, and reference was placed on the inion. (B) During cerebellar stimulation, the anode was placed on the median line 2cm below the inion and reference was placed on the right buccinator muscle.

Twenty-two young adults and twenty older adults volunteered to participate in the study. Two older adults withdrew from the study, one of whom experienced motion sickness and the other dizziness. Participants were excluded based on any medical conditions or medication use that could lead to postural impairment or an adverse reaction to TMS/tDCS. Screening included the use of two TMS pre-screening questionnaires based on Rossi et al.’s [38] guidelines (this includes, but is not limited to, items related to history of seizures, brain injury, metal implants and use of anti-depressants) and a medical questionnaire specific to the postural control task (which includes, but is not limited to, items related to fall history, hip/knee replacement and use of tranquilisers) in accordance with the School of Psychology’s Standard Operating Procedures for TMS. This screening also ensured suitability for tDCS use, as TMS screening and safety guidelines are stricter and better established [39]. Six young adults and two older adults were excluded from analysis as outliers, resulting in a sample of 16 in each age group. The demographic information from this sample is listed in Table 1. All older adults scored 25+ on the *Mini-Mental State Examination (MMSE*; [40]) and showed no impairment in daily function, as assessed by the *Katz Basic Activities of Daily Living* test [41] and the *Instrumental Activities of Daily Living Scale* [42]. Cognitive function was assessed using two subtests from the Wechsler Adult Intelligence Scale (WAIS; [43]), digit symbol substitution and forward/backward digit span. Lower scores in older adults in the digit symbol substitution task reflect normal age-related decline in processing speed. All participants gave written informed consent and the study was approved by the School of Psychology’s Ethics Committee. The three volunteers pictured in Figs 1–3 also gave informed consent for publication of these photographs.

**Table 1:** Sample means and standard deviations (in parentheses).

	**Young Adults**	**Older Adults**
Age	20.81 (2.07)	72.44 (4.03)
Sex (male, female)	6,10	4, 12
Height (cm)	171.38 (10.01)	164.25 (9.51)*
DSS	66.63(9.80)	42.12 (8.58)*
Digit span	18.06 (2.98)	18.56 (2.66)
MMSE	N/A	28.06 (1.39)

Note

* p< .05. DSS: Digit Symbol Substitution, MMSE: Mini Mental State Examination.

### Apparatus

#### Postural control task

Postural control was assessed under sway-referenced conditions using a Smart Balance Master (NeuroCom International, Inc., Clackamas, OR, USA), which comprises an 18”x18” dual ATMI forceplate and movable three-sided surround (Fig 1A). The system recorded Centre of Pressure (COP) trajectories over time, in both medio-lateral (COP-X) and antero-posterior (COP-Y) directions, at a sampling frequency of 100Hz. The platform was sway-referenced during each trial via a servo-controlled motor, which introduced platform tilt in the sagittal plane about the ankle joint axis, in proportion to the participant’s expected centre of mass (COM) sway angle [36]. COM was estimated from the current COP-Y trajectory using a proprietary second-order Butterworth low-pass filter with a cut-off frequency of 0.85 Hz (3,55). Electro-mechanical delays of the system or due to the filter [44] are negligible, approximately 31ms. The mechanical compliance of the support surface to postural sway was determined by the pre-selected *gain factor* of the test. A typical gain factor of 1.0 results in exact coupling between COP-Y movement and the degree of platform tilt (Fig 1B). This prevents any change in ankle joint angle, thus near-eliminating one of the main proprioceptive signals used in postural control [37]. Gain factors larger than 1.0 result in a more compliant support surface, resulting in greater surface rotations and thus greater sway. In the present study, the gain factor was set to 1.0 for older adults and 2.0 for young adults, in order to produce comparable levels of sway in both age groups. Participants wore a safety harness at all times, which doesn’t restrict movements but ensures safety in the event of a loss of stability. In the case of a loss of stability, an extra trial was re-run at the end of the experimental block. Two cases of loss of stability were recorded in the young adult group and six in the older adult group.

#### Transcranial magnetic stimulation

Motor evoked potentials (MEPs) were recorded using single-pulse TMS to examine whether tDCS induced excitability changes in the leg area of the primary motor cortex (M1). TMS was delivered using a Magstim Rapid stimulator attached to an angled double cone coil (Fig 2A; Magstim, Whitland, United Kingdom). Surface electromyography (EMG) signals were recorded from the left and right tibalis anterior muscles using disposable Ag-AgCl electrodes (Cleartrace, CONMED, Utica, NY, USA), with an inter-electrode distance of 3cm and a reference electrode placed on the left lateral malleolus. In order to standardise muscle activity, each participant’s EMG amplitude during maximum voluntary contraction (MVC) was obtained at the beginning of the experiment and they were asked to maintain this contraction at ~20% during each TMS stimulation block [19]. In order to achieve this level of contraction, a foot support with a strap placed dorsally over the foot was used, against which participants were asked to dorsiflex (see Fig 2B). An average of the maximum flexion was taken from the EMG signal and a horizontal cursor marking 20% of this value was placed on the axis on a screen (located ~65cm from the participant), to allow participants to control their flexion using visual biofeedback. TMS during active flexion permits the use of lower TMS output intensities, which promotes compliance during testing with a double cone coil [19].

The motor hotspot corresponding to the tibalis anterior was identified using TMS commencing at the vertex at an intensity of 35% of maximum stimulator output (MSO). This area was gradually explored in steps of 0.5cm and the intensity increased in steps of 1%, until the scalp region exhibiting reliable MEP amplitudes was identified. The position was marked on the scalp with a red dot and the coordinates recorded. The *‘active motor threshold’*, defined as the percentage of MSO required to induce an MEP during 50% of TMS pulses [45], was then identified by adjusting the intensity (MSO) in steps of 1% above the motor hotspot. Once identified, MEP recruitment curves (RCs) were recorded, by measuring the peak-to-peak amplitudes of MEPs as a function of stimulus intensity, ranging, in steps of 5, from 90–130% of the active motor threshold. Stimulus intensities were presented randomly, with seven pulses at each intensity.

#### Transcranial direct current stimulation

tDCS was delivered through two sponge electrodes (anode surface area (SA): 50cm^2^, reference SA: 25cm^2^) soaked in saline solution, attached to a Chattanooga Iontophoresis Dual Channel Delivery Device (Chattanooga Group, Hixson, TN, USA). For both anodal stimulatory conditions, tDCS was set to 2mA for 20 minutes for all participants, resulting in an applied current density of 0.04mA/cm^2^, which is within the safety threshold [46]. This tDCS protocol was motivated from Kaski et al.’s [19] work, which was one of the first papers to demonstrate an effect of tDCS on a lower limb adaptation task. The current was initially ramped up in 0.1mA increments over a 30s period, with an equivalent descending ‘ramping down’ at the end of stimulation. During primary motor cortex stimulation (Fig 3A), the anodal electrode was placed on the leg area motor hotspot, as identified during single-pulse TMS, and the reference electrode was placed on the inion [19]. During cerebellar stimulation (Fig 3B), the anodal electrode was placed on the median line 2cm below the inion [47] and the reference electrode was placed on the right buccinator muscle [22]. Sham stimulation was identical to this but current was only present for the initial 30s ramping up phase and final 30s ramping down phase. This is a standardised sham technique, as the literature reports that naïve participants cannot distinguish between real and sham tDCS [48], as the ‘tingling’ sensation experienced during tDCS typically fades after 10-20s [19]. Half of the participants experienced sham stimulation with the M1 montage and the other half on the cerebellar montage. Participants were asked to report if at any point during testing tDCS became too uncomfortable, in which case the participant would have been withdrawn from the study. However, all participants demonstrated good tolerance to tDCS within the current study.

### Procedure

**Fig 4:**
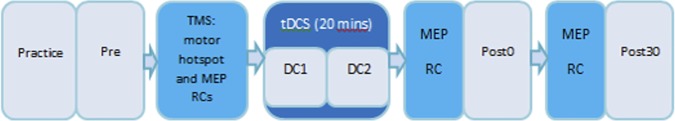
Session schematic. Schematic representation of how the postural control task blocks are separated during each session.

Testing was carried out over three sessions, one for each of the three types of stimulation; sham, cerebellar and primary motor cortex (M1). The study employed a double-blind, repeated-measures design, in which session order was counterbalanced across participants and sessions were separated by 3–7 days. Participants were told that they would experience a different type of stimulation during each session but the details of each stimulation type were not disclosed until debriefing in the end of the final session. Two experimenters were involved in all sessions. The first session lasted approximately 2.5 hours and the other two sessions lasted 2 hours. Each session commenced with a practice block of the postural control task (Fig 4). This block was identical to the five following experimental blocks and familiarised the participants with the sway-referenced platform. Each block consisted of 6 1-minute posture trials; the first 3 trials took place with eyes open, during which participants were asked to fixate on a 3x3cm cross, placed at eye-level ~60cm from them, and the last 3 trials were performed without vision, with participants asked to maintain the same head position to that in the eyes open condition. Trials were performed consecutively, with each trial starting approximately 15s after the previous. During the postural control task, participants were instructed to stand upright on the platform and keep as stable as possible, with their arms by their sides. Participants were not informed about how the platform rotations were generated until debriefing after the final session. After the first experimental block (pre), participants were seated and single-pulse TMS was used to find their motor hotspot and their ‘active motor threshold’. MEP recruitment curves (RCs) were then recorded to assess current corticospinal excitability. This was followed by another two postural control task blocks (DC1 & 2) during which a 20-minute tDCS condition was performed. tDCS was initiated as the postural control task commenced and the two blocks (DC1 & 2) were performed consecutively, with no break offered in between. The primary experimenter left the room before the two blocks commenced and another experimenter entered in order to apply the tDCS stimulation. Thus, the primary experimenter was not aware of the order of tDCS conditions until all participants were tested and the data was analysed. MEP recruitment curves and postural control were assessed again, immediately after tDCS (post0) and 30 minutes after tDCS (post30). Participants were given a seated refreshment break between the post0 and post30 testing blocks.

### Data analysis

Preliminary data pre-processing and analysis was carried out using custom-written Matlab software (The Mathworks, Natick, MA). COP data from each 1-minute trial was low-pass filtered at 4 Hz, using a fourth-order dual-pass Butterworth filter. From this data, our main postural measure, anteroposterior (AP) path length (PL), was calculated. AP PL was determined as the overall distance the COP moved in the AP direction throughout a trial. This is indicative of the overall amount of sway in the AP direction, with a greater value reflecting more postural sway. Peak-to-peak sway amplitude along the AP axis was also examined, as well as the mean power frequency (MPF) of AP sway. Peak-to-peak sway amplitude was calculated as the difference between the maximum and minimum amplitudes of the COP in the AP direction. An increase in this value reflects body sway moving closer to the limits of stability and an increased possibility of a loss of balance. MPF is a measure of the average sway frequency calculated from the spectral power of the COP data. An increase in sway frequency reflects increased production of corrective torque around the ankle joint [37], with higher values indicative of greater joint stiffness and possibly muscle co-contraction [49,50] whereas a decrease in sway frequency reflects reduced production of corrective torque around the ankle joint [37]. MPF was calculated from the Power Spectral Density of each 30s window of the AP COP data using Welch's overlapped segment averaging estimator function (‘pwelch’ in Matlab). For all measures, data from the two blocks (2 and 3) performed during stimulation were averaged. Statistical analyses were carried out using SPSS 21 (IBM Corporation, Armonk, NY). 4-way mixed ANOVAs were calculated for both PL and sway amplitude, with stimulation (sham, M1, cerebellum), block (pre, during stimulation (DC), post0, post30) and visual condition (eyes open/closed) as within-subject factors, and age group as the between-subject factor. The MPF analysis also examined the difference in power frequency within the first 30s of a block compared to the final 30s, thus two 4-way mixed ANOVAs were calculated separately for the eyes open and eyes closed conditions for MPF, with stimulation (sham, M1, cerebellum), block (pre, during stimulation (DC), post0, post30) and time (first 30s vs final 30s) as within-subject factors, and age group as the between-subject factor. Planned contrasts were used to examine significant effects, in order to specifically investigate the differences between anodal stimulation and sham and the differences between pre-stimulation performance and each block following.

The average peak-to-peak amplitude of MEPs for each stimulus intensity were calculated in Matlab and recruitment curves were plotted. From this, the area under the recruitment curve (AURC) was calculated separately for each block in each session using the trapezoidal rule, in line with Carson et al. [51]. A 3x3 mixed ANOVA was computed for within-subject factors, stimulation and block, and the between-subjects factor of age group.

## Results

### Anterior-posterior (AP) path length

**Fig 5:**
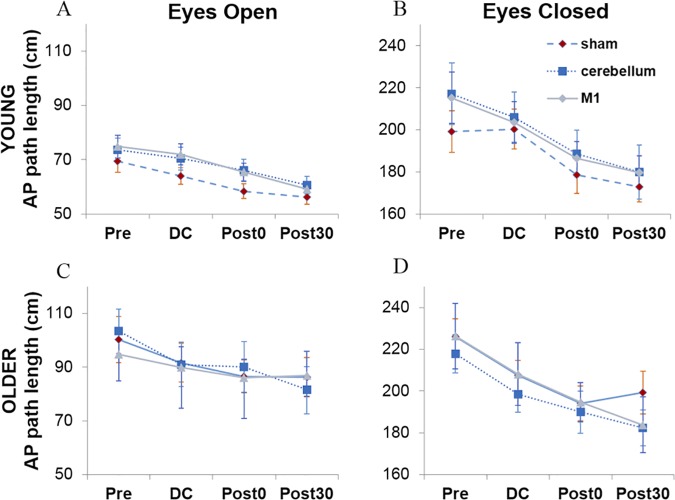
AP path length. AP path length across each testing block and each stimulatory condition for young (A & B) and older adults (C & D) during eyes open and eyes closed respectively. Note the different scale in B and D due to greater sway in Eyes Closed conditions.

Fig 5 depicts the mean AP path length data for each age group for eyes open and closed across each type of stimulation (sham/M1/cerebellum) and testing block (pre, DC, post0 and post30). A 4-way mixed-design ANOVA indicated no significant effects of stimulation condition, *F*(2,60) = .142, *p* = .87, or interactions with stimulation in contrast to our main hypothesis. This suggests that our tDCS manipulation had no effect on AP path length. Participants showed an overall practice effect as shown by a main effect of testing block, $F(1.87,56.07)=39.34,p<.001,\eta_{p}^{2}=.57$. Further exploration using simple planned contrasts, with an alpha value corrected for multiple comparisons to 0.013, revealed a significant difference in path length between pre-test and every other block–DC $F(1,30)=21.49,p<.001,\eta_{p}^{2}=.42$, post0 $F(1,30)=43.14,p<.001,\eta_{p}^{2}=.59$, and post30 $F(1,30)=57.35,p<.001,\eta_{p}^{2}=.66$. Overall, no age differences in path length were shown and no interactions involving age, suggesting that our matching of AP sway path length was successful. As expected, there was a large difference between eyes open and eyes closed conditions, $F(1,30)=710.59,p<.001,\eta_{p}^{2}=.96$ and a block by vision interaction, $F(1.76,52.67)=15.41,p<.001,\eta_{p}^{2}=.34$, which permitted further simple effects analysis.

Separate analyses for each visual condition revealed that there was a larger effect of block in the eyes closed condition, $F(1.83,54.95)=37.99,p<.001,\eta_{p}^{2}=.56$, compared to the eyes open condition, $F(1.83,54.75)=12.81,p<.001,\eta_{p}^{2}=.30$. Planned contrasts, with an alpha value corrected for multiple comparisons to 0.013, revealed that in both visual conditions, a significant decrease in path length from pre-stimulation was found across each block (*p* = .007–.001). Additionally, simple effects analysis of each block showed a significant effect of visual condition across every block (*p* < .001).

### Peak-to-peak sway amplitude

**Fig 6:**
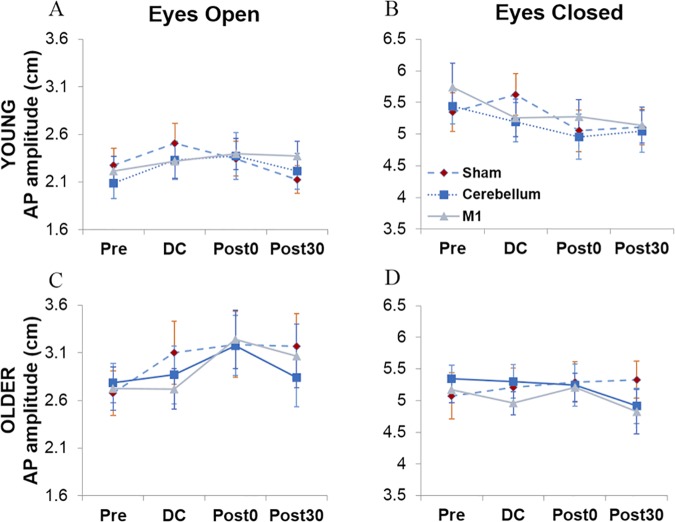
AP peak-to-peak sway amplitude. AP peak-to-peak sway amplitude for young (A & B) and older adults (C & D) during both visual conditions respectively as a function of stimulation condition (sham, cerebellar, M1) and testing block (pre-stimulation, during (DC), after (post0) and 30 minutes after (post30)).

Similarly to the path length data, there were no significant age differences in sway amplitude (Fig 6) overall (*p* = .31), however, there was a significant visual condition by age group interaction, $F(1,30)=10.33,p=.003,\eta_{p}^{2}=.26$, with a significant difference between age groups in the eyes open condition, $F(1,30)=5.52,p=.026,\eta_{p}^{2}=.16$. This suggests that for certain postural measures, creating equivalent levels of sway in both age groups is only possible if visual information is withdrawn. Again, we found no significant effects of stimulation condition, *F*(2,60) = .231, *p* = .79, however, in this case there was a trend towards an interaction between stimulation and block, F(6,180) = 2.10, *p* = .055. Additionally, there were two further interactions between age group and block, $F(3,90)=4.66,p=.004,\eta_{p}^{2}=.14$, and block and visual condition, $F(2,90)=13.50,p<.001,\eta_{p}^{2}=.31$, Due to these significant interactions, simple effects analyses were carried out separately for each age group during each visual condition to examine the different responses across blocks.

During eyes open (Fig 6A and 6C), only older adults, $F(3,45)=6.29,p=.001,\eta_{p}^{2}=.30$, showed a main effect of block. Planned contrasts, with an alpha value corrected for multiple comparisons to 0.013, revealed a significant difference between the pre-test block and post0, $F(1,15)=14.05,p=.002,\eta_{p}^{2}=.48$. Neither group showed a main effect of stimulation. As indicated in Fig 6A, young adults showed similar sway amplitude across blocks, whereas older adults (Fig 6C) showed an increase in sway amplitude until post0. Interestingly, older adults also showed a significant block by stimulation interaction between pre-test and DC for sham compared to cerebellum, $F(1,15)=5.21,p=.037,\eta_{p}^{2}=.26$, and sham compared to M1, $F(1,15)=4.64,p=.048,\eta_{p}^{2}=.24$. During sham stimulation, older adults showed an increase in sway amplitude from pre-stimulation (*M* = 2.68) to DC (*M* = 3.10), however, this is not seen in the anodal tDCS conditions until post0 (*M*_*cere*_ = 3.18; *M*_*M*1_ = 3.24). This suggests that anodal tDCS may delay this increase in sway amplitude in older adults (Fig 6C).

During eyes closed, young adults (Fig 6B) showed a significant main effect of block, $F(3,45)=7.94,p<.001,\eta_{p}^{2}=.35$, which planned contrasts, with an alpha value corrected for multiple comparisons to 0.013, revealed was due to a decrease in sway amplitude from pre-test to post0, *F*(1,15) = 15.61, *p =* .001, $\eta_{p}^{2}$ = .51, and pre-test to post30, $F(1,15)=25.40,p<.001,\eta_{p}^{2}=.63$, however, older adults showed no significant differences across blocks. Both age groups showed no effect of stimulation condition and no stimulation by block interaction.

### Mean power frequency (MPF)

**Fig 7:**
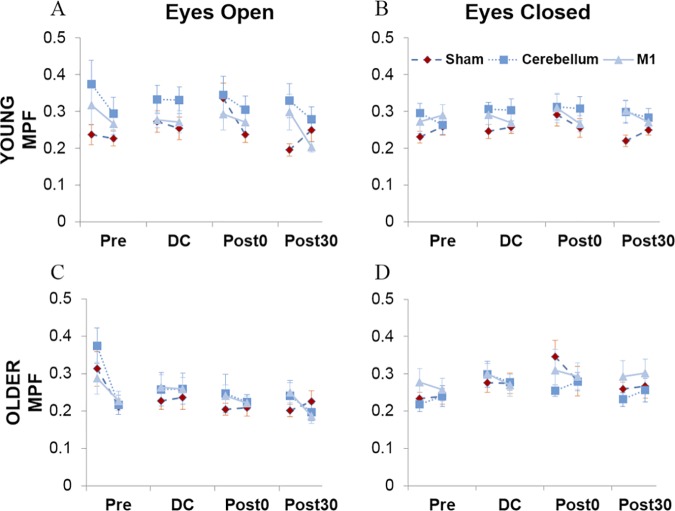
Mean power frequency (MPF). MPF for young (A & B) and older adults (C & D) during each visual condition respectively as a function of stimulation condition (sham, cerebellar, M1), testing block (pre, DC, post0 and post30) and first and last 30s of each testing block.

MPF was analysed separately for eyes open and eyes closed visual conditions (Fig 7). In line with AP path length, neither visual condition showed a main effect of age group (EO *p* = .19; EC *p* = .84). During the eyes open condition, there was an overall main effect of block, $F(3,90)=5.89,p=.001,\eta_{p}^{2}=.16$, which planned contrasts, with an alpha value corrected for multiple comparisons to 0.013, revealed was due to a significant decrease in MPF from pre-stimulation to post30, $F(1,30)=14.19,p=.001,\eta_{p}^{2}=.32$. However, further analysis following a block by age group interaction, $F(3,90)=2.82,p=.044,\eta_{p}^{2}=.09$, revealed that this effect of block was only significant in older adults (*p* = .008). A significant difference between the first and final 30s of each block was also shown for both age groups, $F(1,30)=28.51,p<.001,\eta_{p}^{2}=.49$. Interestingly, there was also a significant effect of stimulation on MPF, $F(1.56,46.8)=5.82,p=.005,\eta_{p}^{2}=.16$, which planned contrasts, with an alpha value corrected for multiple comparisons to 0.025, revealed was due to a significant difference between sham and the cerebellar condition, $F(1,30)=8.40,p=.007,\eta_{p}^{2}=.22$. This effect was dependent on block and whether it was within the first or final 30s of the block, as indicated by a significant 3-way interaction, $F(4.38,131.31)=2.48,p=.042,\eta_{p}^{2}=.08$. Separate analyses for the first and final 30s, with an alpha value corrected for multiple comparisons to 0.025, revealed that the effect of stimulation was only significant in the first 30s of each block $\left(p=.005,\eta_{p}^{2}=.24\right)$. Following this, simple effect analyses examining the difference between sham and cerebellar stimulation for each block during only the first 30s (corrected alpha value = 0.013), revealed that this effect of stimulation during the initial blocks was due to a significant baseline difference between the sham and cerebellar conditions $\left(p=.011,\eta_{p}^{2}=.19\right)$, whereby mean MPF was higher in the cerebellar condition at baseline compared to the sham conditions. This difference was no longer significant in the stimulation (DC) block and immediately following stimulation (post0) but then returned 30 minutes (post30) after stimulation $\left(p=.006,\eta_{p}^{2}=.23\right)$. This suggests that there may be an actual effect of stimulatory condition during this final block, whereby MPF is greater in the cerebellar condition compared to sham.

This interaction between stimulation condition and block was explored further using simple effects analyses to examine the effect of block within the sham and cerebellar condition separately (corrected alpha value = 0.025). In the sham condition, there was a block by age group interaction, $F(1.96,,58.93)=6.64,p=.003,\eta_{p}^{2}=.18$. Whilst both groups showed an effect of block (Young: *p* = .012, $\eta_{p}^{2}=.28;\;Older:\;p=.021,\eta_{p}^{2}=.26$), follow-up Bonferroni post-hoc comparisons revealed that for young adults MPF declines from post0 to post30 (*p* = .011), whereas there were no differences between any blocks for older adults. In the cerebellar condition, there was no effect of block (*p* = .050) when corrected for multiple comparisons (corrected alpha value = 0.025) and no block by age group interaction (*p* = .42). This suggests that for older adults, the effect of cerebellar stimulation on MPF values is caused by a baseline difference between the sham and cerebellar conditions, as there is no difference between blocks in both of these conditions. However, in young adults, during sham there is typically a decline in MPF from post0 to post30, which does not occur following cerebellar stimulation. This suggests that cerebellar stimulation may disrupt the reduction of sway frequency typically witnessed in the final block in young adults and thus prolong postural instability.

During the eyes closed condition there was also a main effect of block, $F(3,90)=6.10,p=.001,\eta_{p}^{2}=.17$, however, in this case, planned contrasts with an alpha value corrected for multiple comparisons to 0.013, revealed it was due to a significant increase in MPF from pre-stimulation to DC, $F(1,30)=11.37,p=.002,\eta_{p}^{2}=.28$, and post0, $F(1,30)=10.61,p=.003,\eta_{p}^{2}=.26$. There were no significant interactions between block and any other variable. Interestingly, during eyes closed, there was no difference between the first and last 30s of each block (*p* = .32). There was no overall effect of stimulation (*p* = .12), however, there was a significant stimulation by age group interaction, $F(2,60)=3.40,p=.040,\eta_{p}^{2}=.10$. Planned contrasts, with an alpha value corrected for multiple comparisons to 0.025, revealed that this interaction was specific to the sham vs cerebellar conditions, $F(1,30)=8.44,p=.007,\eta_{p}^{2}=.22$. However, simple effects analyses for each age group, with an alpha value corrected for multiple comparisons to 0.025, failed to demonstrate a difference between the cerebellar and sham condition for both age groups (Young: *p* = .047; Older: *p* = .21).

### MEP area under the curve

**Fig 8:**
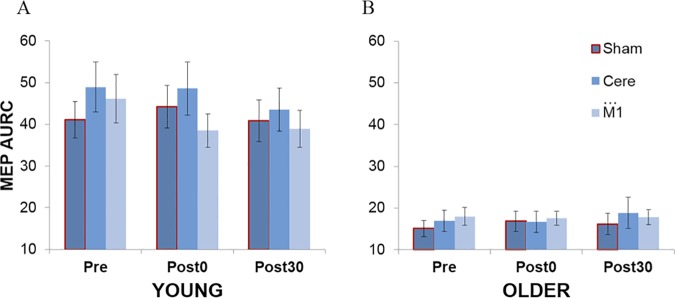
MEP area under the recruitment curve (AURC). AURC averaged across both legs for (A) young and (B) older adults as a function of stimulation condition and testing block.

As there was no significant difference in the corticospinal excitability measured from each leg, statistical analysis was carried out on the averaged data from both legs. A significant effect of age group was found, $F(1,30)=30.24,p<.001,\eta_{p}^{2}=.50$, in which the area under the recruitment curve (AURC) was consistently higher in young adults (*M*_*AURC*_ = 43.37) in comparison to older adults (*M*_*AURC*_ = 17.13), suggesting significantly higher corticospinal excitability in the young group (Fig 8). However, there was no significant effect of stimulation condition (*p* = .19) or testing block (*p* = .35) and there were no significant interactions between any of the factors.

## Discussion

The aim of the current study was to assess whether anodal tDCS of the cerebellum or M1 could affect postural stability in young or older adults. This was tested in a double-blind, sham-controlled design in which postural stability was assessed using a sway-referencing paradigm, which aimed to minimise baseline age differences in postural sway. Effects of stimulation on postural control were minimal and varied greatly between the postural measures, age groups and visual conditions, and there was no effect of stimulation on corticospinal excitability. The minimal effects of stimulation on postural measures were only observed in the eyes open visual condition. For older adults, this was manifested in the sway amplitude measure, as both stimulation conditions (cerebellar and M1) delayed the increase in sway amplitude witnessed between baseline and during ‘stimulation’ (DC) in the sham condition, until the post0 block. This suggests that online anodal stimulation over the cerebellum or M1 prevented the increase in sway amplitude witnessed between the first and second block in older adults, delaying this response until stimulation was no longer active. In contrast, in young adults, minimal effects of stimulation were shown only in the MPF measure, once stimulation was no longer active (off-line), as young adults showed significantly higher MPF in the post30 block in the cerebellar stimulation condition compared to the sham condition. This suggests that cerebellar stimulation deters the typical decline in sway frequency witnessed between post0 and post30 in young adults, thus interrupting the postural practice effect witnessed in these final blocks.

Together, these results suggest that anodal tDCS over the cerebellum or M1 may affect postural control. However, the effects are dependent on the postural measure assessed, the age group, the availability of visual information (eyes open vs closed) and whether the stimulation is currently active or not. Furthermore, it is unclear from our results whether the few effects of stimulation have a positive or negative impact on postural stability. For example, the offline effects of cerebellar stimulation on sway frequency in young adults are likely to be disruptive to postural stability, as this condition impedes the reduction in sway frequency typically witnessed between the final two blocks. However, the delayed increase in sway amplitude witnessed during active anodal stimulation over either the cerebellum or M1 in older adults during eyes open could indicate a positive effect whereby online stimulation causes decreased sway amplitude, which then disappears after stimulation is withdrawn. Alternatively, this could also mean that stimulation interrupts the natural response to introducing a sensory transition, which may later have a negative impact on postural responses. The differences between young and older adults are useful, as they suggest that stimulation had only offline effects on young adults and online effects on older adults. Furthermore, the fact that stimulatory effects were only witnessed in eyes open trials, where postural sway was considerably less, could suggest that the effects of tDCS on postural control were too weak to affect postural responses to more destabilising conditions, such as those during eyes closed conditions. Despite the small effect sizes, post hoc power analyses using G*Power software [52] indicated strong statistical power (Power = .91-.99) for each of the significant stimulatory effects, suggesting that these effects are reliable.

Our main postural control measure, AP path length, showed that both age groups demonstrated a clear practice effect across both visual conditions, in which AP path length decreased across subsequent testing blocks. This is in accordance with previous research from Doumas and Krampe [3], who also showed path length reduction over successive sway-referenced trials. However, the effect of testing block becomes more complex within the other postural measures, which differ between age group and stimulation condition. For example, during eyes open conditions, young adults showed no overall difference in sway amplitude or MPF across testing blocks, whereas, older adults showed an increase in sway amplitude until post0 and a decrease in MPF across blocks. This increase in sway amplitude and decrease in MPF could suggest that older adults switch to an increasing reliance on a hip strategy [53] throughout the blocks. Unfortunately, kinematic measures were not assessed in the present study to clarify this strategy. This difference in strategies is also implied during the eyes closed condition, in which young adults consistently decreased their sway amplitude across blocks, whereas older adults maintained similar amplitude across blocks. However, it is worth noting that in this case young adults started at slightly higher sway amplitude at baseline, which suggests that young adults appear to be more affected by the withdrawal of visual information initially. These complex interactions, which show that the postural response across each testing block is dependent on the measure used to assess posture and the age group it is being assessed in, may explain why we found complex interactions for the tDCS conditions also.

Overall, the complex interactions with tDCS condition may not be surprising considering the multifaceted nature of postural control. tDCS could affect multiple aspects of postural control, including incoming sensory information, the integration of sensory signals (sensory reweighting) or the selected motor outcomes. Furthermore, the lack of effect on corticospinal excitability may not be unexpected, as a recent review of the neurophysiological literature on tDCS [54] showed that studies which have reported an effect on MEPs often found large inter- and intra-subject variability in MEP amplitude, which can result in problems with replication. Further problems of replication occur in tDCS studies due to the lack of knowledge of the localisation of current transfer, which results in difficulties predicting the functional outcomes of tDCS delivered over a specific area [18]. For example, since designing the present study, modelling evidence has emerged that suggests that the previously accepted electrode montages for the M1 are sub-optimal, with the maximum electric field strength occurring in between the two electrode sites rather than directly beneath the anode as originally thought [55]. If we were to replicate this study, we would utilise the new montage that Rampersad et al. [55] suggest, in which the anode is placed 5cm posterior to the motor cortex site whilst the cathode is placed 5cm anterior. Additionally, open-source software has become available that enables users to model the electric field induced by specific electrode montages, such as SimNIBS (http://simnibs.de/version2/start). Improved accessibility of such models will promote the replicability of future studies.

Our current study protocol was motivated by previous research by Kaski et al. [19], however, there are several modifications we would make to future protocols. Firstly, we would ask participants to self-report their sensation of the stimulation during each session, in order to monitor the success of the blinding procedure. Secondly, if using a standard bipolar montage, we would reduce the electrode size in order to improve focality and increase current density [56,57]. Specifically, we would reduce the size of the anodal electrode to ~3.5cm^2^ and increase the size of the reference electrode to 35 cm^2^ [57]. Alternatively, a more recent technique, namely multifocal tDCS, would further optimise the electric field [58]. This technique utilises multiple small electrodes configured around a site to achieve greater focality. Furthermore, this technique can also be used to stimulate multiple cortical sites simultaneously, thus potentially enabling the modulation of specific brain networks [58]. Consequently, such techniques would be highly relevant to postural control and/or sensory reweighting, both of which are hypothesised to involve distributed networks within the brain. For example, future research could investigate whether combined stimulation of the primary motor cortex alongside the sensorimotor cortex could further improve sensory integration for postural control. Previous research has shown that 20 minutes of 2mA tDCS over the left sensorimotor cortex could increase neural responsiveness to foot pressure stimuli in healthy young adults [59]. Decline in proprioceptive [60,61] and cutaneous sensitivity [62,63] has been associated with reduced postural control in older adults. Consequently, stimulation of somatosensory regions alongside motor regions could improve postural outcomes in older adults.

Another interpretation of our minimal effects of tDCS could be that this type of continuous posturography measure may be too variable to see distinct effects of tDCS. Previous studies which have reported significant effects of anodal motor/premotor tDCS on postural outcomes have employed more discrete measures, such as the response to a specific perturbation [33,34]. Additionally, the current study was limited in that it only assessed postural measures in the AP direction. Previous research has indicated that mediolateral (ML) sway may be particularly relevant in differentiating older adults at risk of falls [63]. Therefore, future studies could compare the effect of tDCS on AP and ML sway in older adults in response to a perturbation. Duarte and colleagues [35] reported significant improvement in stabilometric measures after anodal primary motor tDCS and treadmill training in children with cerebral palsy. However, they employed multiple tDCS sessions, during which children underwent 20 minutes of treadmill training whilst receiving 1mA anodal tDCS, five times a week for 2 weeks. Additionally, each of these previous studies assessed postural outcomes in clinical populations, so it is possible that the healthy older adults who participated in our study were performing too well at baseline to see similar benefits of tDCS.

On the other hand, dual-task scenarios present additional postural challenge in older adults [64–67], which could lead to more demonstrable effects on postural outcomes. This has been shown by Manor and colleagues [68], who reported that anodal 2mA tDCS over the prefrontal cortex (PFC) led to significantly lower dual-task costs when concurrently performing a serial-subtraction task during standing or walking in healthy older adults. However, in this case, lasting effects of tDCS were not assessed, thus it is unclear whether this intervention could have long-term implications on older adults’ postural control during dual-task scenarios. Furthermore, it is important to note that tDCS did not alter single task performance. The authors postulate the neural effects of tDCS may have led to increased ‘cognitive reserve’, resulting in improved ability to perform both tasks concurrently. Similar results were reported by Zhou et al. [69] for anodal 1.5mA tDCS applied for 20 min over the left dorsolateral PFC in healthy young adults. This may be important for real-life postural control, which often requires individuals to complete multiple other tasks whilst maintaining stable upright stance. The effect of dual-task conditions on the postural response to sway-referencing has been assessed previously by Doumas and colleagues [70,71], however, future studies could investigate how these responses are affected by tDCS. Additionally, future studies could examine the effect of tDCS on an alternative postural task, other than sway-referencing, in order to clarify whether the minimal effects witnessed were due to a modulation of sensory reweighting or another postural mechanism. Also, a larger sample of older adults could have enabled a comparison between the effects of tDCS on a ‘younger’ older adult (aged 60–70) and an ‘older’ older adult (aged 70+) population. Previous authors [72] have noted that stimulation effects may not be present in patient groups due to neurological changes. Similarly, tDCS may affect an older population (over 75) differently due to age-related neurological changes.

In conclusion, the present study suggests that anodal tDCS over the cerebellum or primary motor cortex has minimal effects on postural control which are dependent on the postural measures assessed and have complex interactions with the age group and visual condition (eyes open/closed). Furthermore, it is unclear from the current findings whether these stimulation effects will have a positive or negative impact on postural stability overall. Other research has shown that anodal stimulation over prefrontal areas can have a positive impact on dual-tasks costs during quiet stance in older adults [68], however, this research focuses more on the cognitive resources required for postural control and multitasking, rather than sensory integration, which was the focus of this study. It is possible that tDCS cannot target sensory integration processes, as these may be lower level processes, utilising subcortical networks. Previous tDCS studies have shown success with clinical groups, using discrete measures and/or utilising multiple tDCS sessions [30,32–35]. It may be that the effects of tDCS are too discrete to witness clearly in a complex, dynamic response, such as that of posturography measures.

## Supporting Information

S1 Data Zip file of pre-processed SPSS data. (ZIP)
